# The ATENción Plena en Enfermedad de Alzheimer (ATENEA—Mindfulness in Alzheimer’s Disease) Program for Caregivers: Study Protocol for a Randomized Controlled Trial

**DOI:** 10.3390/healthcare10030542

**Published:** 2022-03-15

**Authors:** Alicia Sánchez-Pérez, Daniel Mendialdua-Canales, Miriam Hurtado-Pomares, Paula Peral-Gómez, Iris Juárez-Leal, Cristina Espinosa-Sempere, Paula Fernández-Pires, Inmaculada Zango-Martín, Inmaculada Abellán-Miralles, Pablo López-González, Desirée Valera-Gran, Eva-María Navarrete-Muñoz

**Affiliations:** 1 Department of Surgery and Pathology, Miguel Hernández University, 03550 Alicante, Spain; alicia.sanchez@umh.es (A.S.-P.); mhurtado@umh.es (M.H.-P.); pperal@umh.es (P.P.-G.); ijuarez@umh.es (I.J.-L.); c.espinosa@umh.es (C.E.-S.); paula.fernandezp@umh.es (P.F.-P.); enavarrete@umh.es (E.-M.N.-M.); 2 Grupo de Investigación en Terapia Ocupacional (InTeO), Miguel Hernández University, 03550 Alicante, Spain; 3 Alicante Institute for Health and Biomedical Research (ISABIAL-FISABIO Foundation), 03010 Alicante, Spain; 4 Order of Interbeing, 03580 Alfaz del Pi, Spain; danielmendialdua@gmail.com; 5 Occupational Therapy Department, University School of Nursing and Occupational Therapy of Terrasa, Autonomous University of Barcelona, 08221 Terrassa, Spain; indazango@euit.fdsll.cat; 6 Behavioural Neurology and Dementia Unit, Hospital San Vicente del Raspeig, 03690 San Vicente del Raspeig, Spain; iabellanm@gmail.com; 7 Asociación Alzheimer de Alicante, 03016 Alicante, Spain; gerencia@alzheimeralicante.org

**Keywords:** mindfulness, caregiver, Alzheimer’s disease, depression, anxiety, neuropsychiatric symptoms, occupational performance

## Abstract

A person affected by Alzheimer’s disease (AD) gradually loses the ability to perform activities of daily living and becomes dependent on caregivers, thereby having a negative impact on the caregivers’ quality of life. There is evidence that suggests that interventions aimed at caregivers, such as mindfulness, may be effective at reducing this burden and emotional issues, such as depression and anxiety, and improving their quality of life. However, there is a lack of consistency in the findings and conclusions remain tentative. In addition, as neuropsychiatric symptoms (NPSs) of AD are major determinants of the caregiver’s burden, these interventions should examine the relationship between these symptoms and caregiver outcomes. Importantly, to improve the design of therapeutic interventions for caregivers and complement the treatment of AD, aspects related to occupational performance and the participation of people with AD and their caregivers should also be considered. Therefore, this study will aim to examine first, the effects of a mindfulness-based program designed for caregivers on NPSs of AD and caregivers’ anxiety and depression; second, the effects of this program on patients’ functional capacity, cognitive performance, executive functions, and quality of life, and on caregivers’ burden, quality of life, occupational balance, executive functions, psychological wellbeing, and self-compassion. We believe that the findings of this study will have significant implications for future healthcare strategies focused on improving the quality of life and wellbeing of caregivers.

## 1. Introduction

Dementia is one of the main causes of death, disability, and dependency among the older population; thereby, it remains a public health issue of high priority [1]. Alzheimer’s disease (AD) is the most common type of dementia and, globally, it accounts for around 60–70% of the cases according to the latest estimates [2]. AD is characterized by a progressive loss of neurons that clinically manifests itself in a gradual decline of cognitive functions and in the emergence and worsening of neuropsychiatric symptoms (NPSs) [3]. Both cognitive decline and NPSs seem to be directly responsible for the deterioration in the functional capacity of the person affected by AD, who gradually loses the ability to perform activities of daily living and becomes alienated from his/her surroundings and dependent on caregivers [4].

It is well known that AD has a negative impact on caregivers’ quality of life not only in economic terms, but also constitutes a major threat to their mental and physical health and social wellbeing [5,6,7,8]. Although the need for supervision and demand for higher care resulting from the impairment of cognitive functions due to AD have been linked to high levels of strain in caregivers, accumulating evidence suggests that NPSs are likely to be the main determinants of the caregiver’s burden [9,10,11,12,13]. However, due to the lack of consistency in the methodological approach of studies, it remains unclear which NPSs may cause the most negative outcomes for caregivers. In contrast, regardless of the types and severity of NPSs, evidence does seem to support that caregivers’ depression and perceived stress are closely related to both NPSs and caregivers’ burden [14]. Since depression and perceived stress may increase the feeling of burden [10,11,12,13,14] and provoke caregiving problems, such as difficulty in controlling NPSs or using inappropriate coping skills [14], studies focused on identifying and managing mental health disturbances in caregivers are clearly warranted.

Mindfulness, among others, is one of the most common nonpharmacological interventions aimed at caregivers. Mindfulness is generally considered a practice involving consciously paying attention to the experience of the present moment with interest, curiosity, and acceptance [15]. In this respect, the overall goal of a mindfulness-based program designed for caregivers is to enable them to cultivate an experiential understanding of their bodily sensations, feelings, thoughts, and impulses, resulting in self-examination and better self-understanding of their daily living, especially while performing the activity of caregiving. A recent meta-review based on systematic reviews and meta-analyses of nonpharmacological interventions for informal dementia caregivers showed that mindfulness-based interventions may be effective for caregivers in reducing burden and emotional issues, such as depression and anxiety, as well as in improving their quality of life [16]. However, evidence remains tentative mainly due to concerns about methodological issues, including those related to study design, sample size, and follow-up, as well as about the heterogeneity of the study findings. In addition, one important aspect lacking in most of the mindfulness-based intervention studies is determining the relationship between the patient symptoms, including NPSs, and caregiver outcomes. To the best of our knowledge, this has still not been fully examined. In parallel, since occupational performance issues directly affect, although differently, both the people with AD and their caregivers by affecting their roles, habits, and routines [17,18,19,20], it is also interesting to know if mindfulness-based interventions have a positive impact on people with AD and their caregivers in terms of occupational functioning. Although incipient, recent evidence has shown that nonpharmacological interventions developed using an occupational-based approach are promising strategies for reducing NPSs in people with AD and to minimize caregivers’ burden [17,18,21,22]. Thus, occupational-related outcomes, such as performance skills, in combination with global health measures (e.g., quality of life, wellbeing, and occupational balance) are crucial factors in monitoring the success of these palliative-type care interventions. Therefore, this study will aim to examine first, the effects of a mindfulness-based program designed for caregivers on the NPSs of people affected by AD and on their caregivers’ anxiety and depression; second, the effects of this program on patients’ functional capacity, cognitive performance, executive functions, and quality of life, and on caregivers’ burden, quality of life, occupational balance, executive functions, psychological wellbeing, and self-compassion.

## 2. Materials and Methods

### 2.1. Study Design and Participants

**Figure 1:**
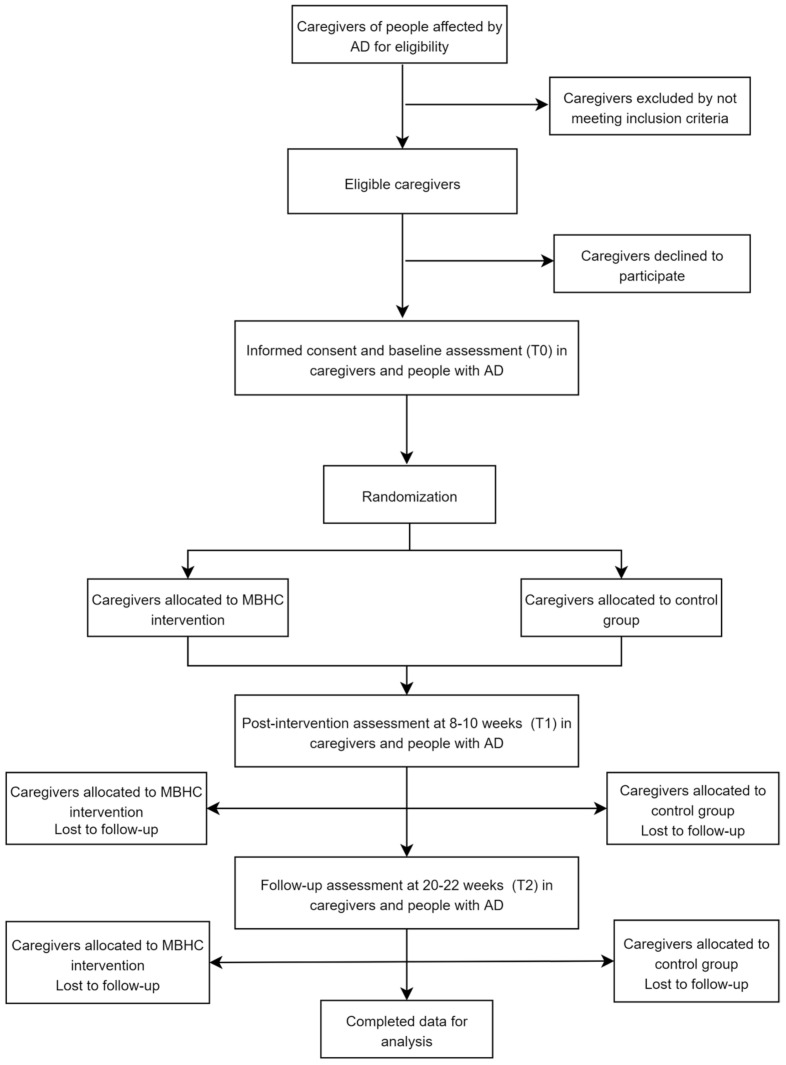
Flowchart of participants in the ATENEA project. Abbreviations: AD, Alzheimer’s disease; MBHC, Mindfulness-Based Health Care.

The ATENción plena en Enfermedad de Alzheimer (ATENEA—Mindfulness in Alzheimer’s disease; http://inteo.edu.umh.es/en/atenea/) project is a randomized, single-blind, parallel group clinical trial for caregivers of AD patients. This trial will include an intervention group that will attend a Mindfulness-Based Health Care (MBHC) program and a control group that will continue with their usual routine. Participants will be caregivers of people diagnosed with mild cognitive impairment due to AD or dementia due to probable AD according to the criteria of the National Institute on Aging and the Alzheimer’s Association (NIA-AA) [23]. The participant assessments of both caregivers and people with AD will be carried out in person. Three assessments will be conducted: at the beginning of the study (baseline assessment T0), at the end of the program at 8–10 weeks (post-intervention assessment T1), and 20–22 weeks after the start of the study (follow-up assessment T2). Figure 1 summarizes the flow chart of the ATENEA project.

### 2.2. Procedure and Enrolment

Participants will be adults from the Alicante province, Spain. People affected by AD and their caregivers will be recruited from the Behavioural Neurology and Dementia Unit of the San Vicente Hospital, General University Hospital of Alicante, and University Clinic Hospital of San Juan; the primary health centers in Santa Pola, San Juan, and Muchamiel; and from four local associations of relatives of Alzheimer’s patients in the province of Alicante. To optimize this process, a massive advertising campaign will also be launched using social networks and posters containing the study information.

The inclusion criteria for caregivers will be: (1) being an informal caregiver of a person diagnosed with mild cognitive impairment due to AD or dementia due to probable AD according to the NIA-AA criteria; and (2) obtaining a score on the Mini-Mental Cognitive Examination (MMSE) equal to or higher than 26. Both caregivers and persons with AD will be excluded if they present: (1) neurological disease history (e.g., stroke, transient ischemic attack, epilepsy, meningitis, etc.); (2) alcohol and drug abuse, excluding tobacco, during the 24 months prior to the start of the study; (3) a systemic disease associated with cognitive impairment; (4) a severe psychiatric illness (major depression, schizophrenia, etc.); and (5) visual and/or auditory perceptual disorders that limit the performance of the tests.

Participants will be assigned by simple randomization to the intervention group (MBHC program) or to the control group (no intervention). Randomization will be carried out by generating a random sequence with the randomizeR package in R statistical software [24]. To control for potential selection bias, personnel from the research team external to the evaluations and the intervention program will be in charge of generating the random sequence and informing the participants by telephone about the group to which they will be assigned to one week before the start of the program. To limit potential information biases, both the evaluators and the professionals conducting the MBHC program will be unaware of the assignment of the study subjects and the results of the evaluation, respectively.

### 2.3. Intervention: Mindfulness-Based Health Program

The MBCH program includes meditative practices using a Buddhist approach [15,25]. This program is mainly focused on practicing several important aspects: (1) paying attention to the present moment; (2) cultivating the acceptance of and openness to the present experience without resistance and avoiding any judgement; (3) developing and strengthening wholesome qualities, such as kindness and compassion; and (4) improving deeper self-inquiry by examining the subjective experience through thoughts, feelings, and sensations. To provide a supportive learning environment and facilitate communication with participants, group dynamics based on person-centered facilitation techniques [26] and non-violent communication practices [27] will be used in each session of the program.

The program consists of eight weekly two-hour sessions, plus an eight-hour silent retreat day. As proposed in a previous similar mindfulness-based program for dementia caregivers [28], the retreat session will not be carried out in the present study. It is assumed that this activity could decrease adherence to the program because of the burden involved in the caregiving role. One week prior to the start of the program, caregiver participants will attend a one-hour briefing session that will explain the content of the program and answer any questions they may have.

The general outline of the MBCH program for caregivers is shown in Table 1. Briefly, all sessions will cover the following set of activities: formal meditation practice (meditation with movement, body-centered meditation, mindful observation, visual concentration, and affect-centered meditation); informal meditation practices (exercises to incorporate mindfulness into daily life); stretching exercises; sharing personal experiences and thoughts; and exercises to do at home. In the event that any activity programmed for a specific session is omitted, this will be included in the following session. Participants will receive printed material on the programmed activities for each session, and an audio guide to performing and recording the exercises to do at home. An example of one of the sessions presented in this printed material is available on the InTeO research group website (http://inteo.edu.umh.es/atenea/ejemplo-de-sesion-de-mindfulness/).

Based on previous studies [29,30,31,32,33,34,35], we propose that the size of each group attending the MBHC program should be between 8 and 15 participants. This group size is considered an adequate number to maintain sufficient group interactions between the facilitator and all participants [28]. Moreover, the program will be repeated by the same instructor in order to minimize variations in the intervention.

To evaluate the implementation process of the program (i.e., adherence to the training protocol), an external observer will take an attendance register of all caregivers in the intervention group and measure their progress and degree of success in performing the session activities using a checklist of the points to consider. Moreover, all caregivers in the intervention group will be asked to complete a satisfaction survey at the end of the program (Supplementary Material File S1). To avoid loss to follow-up, caregivers allocated to the intervention group will be offered a care service if they are accompanied by some relative (e.g., AD patient, disabled relative or child) when attending the MBHC program sessions.

### 2.4. Study Variables

Information about sociodemographics, health issues, and aspects related to the caregiving of participants will be collected using several measurements based on previous studies and different ad hoc questionnaires. Table 2 shows all the information that will be collected during the ATENEA study.

#### 2.4.1. Main Outcomes

##### Caregiver Anxiety and Depression

Both the anxiety and depression symptoms in caregivers will be measured using the Spanish version of the Hospital Anxiety and Depression (HAD) scale [36]. It is a self-administered scale that can be used for assessing the emotional responses of anxiety and depression. Caregivers will be asked to report their feelings experienced during the last week. HAD consists of 14 items split into 2 independent 7-item subscales: a depression subscale and an anxiety subscale. Each item can be scored on a Likert-type scale of 3 points with 4 response options, ranging from 0 to 3. The total score of each subscale is obtained by adding the points obtained from each item, with a maximum score of 21 points for each subscale. In both subscales, scores ≤ 7 indicate no symptoms, scores from 8 to 10 are indicative of unclear symptoms, and scores ≥ 11 identify a case.

##### Neuropsychiatric Symptoms (NPSs) in AD

The presence of NPSs of participants affected by AD will be assessed using the abbreviated Spanish version of the Neuropsychiatric Inventory Questionnaire (NPI-Q) [37]. The NPI-Q assesses both the neuropsychiatric syndromes that affect a person suffering from AD or other neurological disorders over the previous month and the emotional distress experienced by his/her caregiver because of these symptoms. Designed to be self-completed by the caregiver, this questionnaire is divided into 12 neuropsychiatric domains (delusions, hallucinations, agitation/aggression, depression/dysphoria, anxiety, euphoria, apathy/indifference, disinhibition, irritability/lability, aberrant motor activity, sleep/night-time behavior disturbances, and appetite and eating changes). Each NPI-Q domain includes a screening question indicating the main symptoms of that domain to help the caregiver to identify a particular behavior at first. Afterwards, the caregiver will be asked to indicate whether the NPS is present and rate the severity of the symptoms on a scale of 0 to 3 (0 = not present; 1 = mild; 2 = moderate; and 3 = severe). By adding up all the respective points, we will obtain a presence and severity NPI-Q score that ranges from 0 to 36 points. Moreover, the caregiver will also be asked to rate the impact of the symptoms on themselves on a scale of 0 to 5 (0 = no distress; 1 = low distress; 2 = mild distress; 3 = moderate distress; 4 = severe distress; and 5 = extreme distress). The caregiver distress score can be obtained by adding up all the points, which ranges from 0 to 60.

#### 2.4.2. Other Outcome Measures

##### Global Deterioration Scale (GDS)

The severity of the impairment caused by AD will be assessed using the Global Deterioration Scale (GDS) [38]. The GDS was designed to give caregivers/clinicians an outline of the stages of cognitive decline for people affected by dementia, such as AD. The scale can be used to classify cognitive impairment into 7 clinical stages: stage 1 (no cognitive decline), stage 2 (very mild cognitive decline), stage 3 (mild cognitive decline), stage 4 (moderate cognitive decline), stage 5 (moderately severe cognitive decline), stage 6 (severe cognitive decline), and stage 7 (very severe cognitive decline).

##### Mini Mental State (MMS)

The global cognitive function of AD participants will be evaluated using the Mini Mental State (MMS) for the Spanish population [39]. The MMS is a widely used test to monitor the progression and severity of cognitive dysfunction in AD. This is a brief test consisting of several tasks that assess different cognitive functions: orientation, registration, concentration, attention, numeracy, verbal learning, naming, and visuoconstruction. The total score ranges from 0 to 30 and can be obtained by adding up the points for each task. Lower scores on the test imply higher cognitive dysfunction.

##### Frontal Assessment Battery (FAB)

The executive functions of participants with AD and their caregivers will be examined using the Spanish version of the Frontal Assessment Battery (FAB-E) [40,41]. The FAB is a brief test that evaluates the processes controlled by frontal lobe functions. It consists of six subtests: conceptualization, mental flexibility, motor programming, sensitivity to interference, inhibitory control, and environmental autonomy. Each subtest can be rated on a scale of 0 to 3. To obtain the total score, the points for each subtest are added up, providing a total that ranges from 0 to 18 points.

##### Disability Assessment for Dementia (DAD)

The functional capacity of participants with AD will be assessed by the Spanish version of the Disability Assessment for Dementia (DAD-E) scale [42,43]. The DAD is an instrument designed to measure functional disability through the assessment of activities of daily living (ADL) in people with dementia. It evaluates in each ADL whether the person has. the initiative to carry out the activity, if he/she is able to plan or organize it, and if he/she is able to execute it. This scale consists of 40 items that assess basic ADLs (hygiene, dressing, continence, and eating), instrumental ADLs (meal preparation, telephone use, going out, finances and correspondence, medication, and household activities), and leisure activities. The total score, which ranges from 0 to 40, should be converted to percentage terms. Higher percentage scores indicate a better competence in ADL.

##### Caregiver Burden

Caregiver burden will be measured using the Spanish version of the Zarit Burden Interview (ZBI) [44,45]. The ZBI provides an objective measure of caregiving burden, including several aspects, such as consequences of caregiving, patient’s dependence, exhaustion and uncertainty, guilt or self-criticism, embarrassment/anger or frustration, psychological burden and emotional reactions, personal strain, and role strain. The scale consists of 22 items that can be rated on a 5-point Likert scale ranging from 0 (never) to 4 (nearly always). Higher scores on the scale imply a greater burden, and the total maximum score that can be obtained in the ZBI is 88.

##### Quality of Life

In this study, the quality of life for both AD participants and their caregivers will be assessed. To evaluate the quality of life for caregivers, we will use the Spanish version of the SF-36 health survey questionnaire [46]. The SF-36 questionnaire is a self-administered measure consisting of 36 Likert-type items, which takes between 5 and 10 minutes to complete. The questionnaire contains 35 items that measure health on 8 domains (physical functioning (10 items), social functioning (2 items), physical problems (4 items), emotional problems (3 items), mental health (5 items), vitality (4 items), pain (2 items), general health perception (5 items)) and 1 item that evaluates the change in respondents’ general health status from the previous year. To calculate the total score, all item scores will be summed and transformed into a scale ranging from 0 (poor health) to 100 (optimal health).

The Spanish version of the Quality of Life-Alzheimer’s Disease Scale (QoL-AD) [47] will be used to assess the quality of life of AD participants. The QoL-AD will be completed by both the AD participants and their caregivers. This instrument is a 13-item questionnaire that measures the following aspects: physical health, energy levels, mood, living situation, memory, relationship with friends, relationship with family members, relationship with spouse, self-esteem, ability to do chores around the house, ability to do things for fun, financial situation, and life in general. Each item can be rated on a scale from 1 (poor) to 4 (excellent). The QoL-AD scores can be calculated by adding up the points for each item, which range from 13 to 52. To obtain the total score, the AD patient score and the caregiver score should be calculated as follows: ((AD patient score × 2)  + caregiver score)/3. Higher scores on the questionnaire indicate a better quality of life.

##### Occupational Balance Questionnaire (OBQ)

Caregivers’ occupational balance will be measured using the Spanish version of The Occupational Balance Questionnaire (OBQ-E) [48]. The OBQ is a short instrument that assesses occupational performance according to the person’s satisfaction with the amount of and variation in occupation. The questionnaire contains 13 items that can be scored on a scale of 0 (completely disagree) to 5 (completely agree). The sum of the values of each item provides the total score, which ranges from 0 to 65 points. Higher scores are indicative of a better occupational balance.

##### The Self-Compassion Scale (SCS)

The Spanish version of the Self-Compassion Scale (SCS) [49] will be used to measure caregiver’s clinical outcomes resulting from the MBHC program. The SCS consists of 26 items that evaluate 6 components of self-compassion: self-kindness (5, 12, 19, 23, 26 items), self-judgment (1, 8, 11, 16, 21 items), common humanity (3, 7, 10, 15 items), isolation (4, 13, 18, 25 items), mindfulness (9, 14, 17, 22 items), and over-identification (2, 6, 20, 24 items). Each item can be rated on a scale of 1 (almost never) to 5 (almost always). To compute a self-compassion score, items included in the self-judgement, isolation, and over-identification subscales should first be reverse rated. Then, the means for each and all the subscales should be calculated. Higher SCS scores indicate higher self-compassion.

##### The Psychological Well-Being Scale (PWBS)

Caregiver psychological well-being will be assessed using the shortened 29-item version of the Psychological Well-Being Scale adapted to the Spanish population [50]. The PWBS is a self-report measure containing 6 scales: self-acceptance (1, 7, 19, 31 items), positive relations with others (2, 8, 14, 26, 32 items), autonomy (3, 4, 9, 15, 21, 27 items), environmental mastery (5, 11, 16, 22, 39 items), purpose in life (6, 12, 17, 18, 23 items), and personal growth (24, 36, 37, 38 items). Each item can be rated on a Likert-type scale of 1 (totally disagree) to 6 (totally agree). To create a total score, items that are negatively worded should first be reverse coded. Higher scores reflect a high sense of well-being.

### 2.5. Data Management

All study information data will be keyed into Microsoft Office Excel (Microsoft Corporation, Redmond, WA, USA) spreadsheets to create a database. A unique identification number will be used to anonymize confidential personal information on each participant. The principal investigator (A.S.-P.) will be responsible for ensuring appropriate data management and storage, involving a back-up copy of electronic files that will be maintained and stored on a hard drive, and an archive of all the original documents (i.e., questionnaires, tests, and personal data) that will be properly stored in numerical order in binders. Study data accessibility will be restricted, although data will be available on request. All requests will be reviewed by the principal investigator (A.S.-P.) and the research team and will require a data transfer agreement.

### 2.6. Statistical Analysis

#### 2.6.1. Sample Size

The sample size was estimated separately for the AD participants and their caregivers based on the expected effects on the primary outcomes (i.e., anxiety and depression in caregivers measured by the HAD scale and NPSs in AD participants measured by NPI-Q). To calculate the sample for both groups, a bilateral alpha error of 5% and a power of 80% were established. For caregivers, we considered the values provided by the study by Joling et al. [51] assuming a sample size based on an estimated effect of a 10% reduction in anxiety and depression problems. Thus, the sample size for caregivers was 124 participants (62 pairs). For AD participants, a mean difference of 10 and a standard deviation of 15 points were taken as reference values based on previous mindfulness interventions with this clinical population [52,53], thereby obtaining a sample size of 74 people with AD (37 pairs). The final sample was calculated based on the best estimate obtained with the highest number of participants. Furthermore, to counteract the lack of statistical power due to possible loss of follow-up, a 15% of loss was assumed by applying the formula 2 × 62 × (100 ÷ 85) = 145. Therefore, a total of 73 pairs of people with AD and their caregivers need to be recruited. All sampling estimates were performed using the Epidat statistical and epidemiological analysis program (version 4.2), available at www.sergas.es/Saude-publica/EPIDAT (accessed on 20 March 2020).

#### 2.6.2. Data Analysis Plan

Statistical analysis will be performed using the software R, version 4.1.1 (R Foundation for Statistical Computing, Vienna, Austria; http://www.R-project.org). All statistical tests will be bilateral, with the significance level set at 0.05. All data analysis will be conducted using an “intention-to-treat” approach to ensure the initial comparability between groups as obtained by randomization, thereby reducing potential biases.

The general characteristics of the study participants will be described as frequencies and percentages (categorical variables) and as a mean and standard deviation when normally distributed, or a median and interquartile range when not normally distributed (quantitative variables). The distribution of quantitative variables will be assessed using the Lilliefors-corrected Kolmogorov–Smirnov test.

To explore the differences between the intervention and control groups in regard to the main and secondary variables, we will use the Chi-square test or Fisher’s Exact test for categorical variables, and the Student’s t-test or Mann–Whitney U-test for continuous variables. To control confounding bias, bivariate regression models will be used to assess the effect on primary outcomes between study groups using all the significant covariates (*p* < 0.20) to build the core models. Moreover, following a backward elimination procedure, all the covariates associated with the main outcomes will be included at a level of *p* < 0.10. The previous variables, although not statistically significant, will be kept in the models if they change the magnitude of the main effects by more than 10%. Finally, to evaluate the effect of the MBHC intervention on primary and secondary outcomes at baseline, 8–10 weeks (post-intervention), and 20–22 weeks (follow-up), multiple regression models will be estimated.

### 2.7. Ethical Approval, Ethical Considerations, and Dissemination

This study is registered in ClinicalTrials.gov (identifier NCT03858283) and has obtained the approval of the Research Ethics Committee of the Hospital Universitario San Juan de Alicante (code: 18/317), the Universidad Miguel Hernández (registration 2017.413.E.OEP;2017.470.E.OEP), and the Hospital General Universitario de Elche (code 44/2019). The study complies with the Declaration of Helsinki agreement and the official regulations currently in force. Prior to inclusion in the study, all participants will receive verbal and written information about the project, with the contact details of the principal investigator (A.S-P.) and will be asked to sign an informed consent form. Participants will not receive any type of compensation for their collaboration.

For ethical reasons, once the study is completed, the control group will be asked to participate in a MBHC program free of charge. When the study is completed, participants will be informed of the results. We also expect that the results will be published in peer-reviewed journals, international congresses, and the InTeO research group’s website (http://inteo.edu.umh.es/en/atenea/).

## 3. Discussion

This randomized clinical trial has been designed to assess the effect of a mindfulness-based program (MBHC) on caregivers’ symptoms of anxiety and depression, and on NPSs of people with AD. Although there are previous similar mindfulness-based intervention studies aimed at dementia caregivers, to our knowledge, this is the first time that an intervention of this type will explore in parallel emotional disturbances in caregivers, such as anxiety and depression, and the presence of NPSs in people affected by AD. Importantly, since NPSs of AD have been found to be major determinants of caregivers’ burden [9,10,11,12,13], this study may also represent a significant step in determining the relationship between these AD symptoms and caregiver outcomes. Moreover, one marked difference between earlier mindfulness-based intervention studies focused on AD caregivers and ours is that the present study was conceived by adopting an occupation-based research approach. In this respect, we also intend to examine the short- and medium-term effects of a MBHC program on the aspects of both caregivers and AD people that are related to their occupational performance and their degree of autonomy. Considering the great psychological and emotional distress experienced by caregivers and the progressive disturbed functioning of the AD population [17,18,19,20], we will evaluate a wide variety of outcomes to elucidate the impact of the MBHC program on their occupational performance and satisfaction with the purpose of improving meaningful participation in their daily living. As such, from the caregivers’ perspective, we will examine their subjective burden, quality of life, occupational balance, executive functions, and psychological wellbeing; and from the AD people’s perspective, their functional capacity, cognitive performance, executive functions, and quality of life.

In our study, we want to contribute to the evidence showing that mindfulness-based interventions can be effective at reducing the burden and emotional issues for AD caregivers. As mindfulness itself is a complex process to assess, the term “self-compassion” operationalized as the SCS has been proposed as a better indicator of mindfulness for measuring clinical outcomes, such as anxiety or depression [54]. Thus, in this study, we hope, using the SCS as a valid and accurate measure of mindfulness, to provide more convincing evidence about mindfulness-based interventions as a promising approach to helping caregivers of people with AD. In this sense, if the MBHC program designed for this study is effective, one of its advantages is that its applicability can be extended to healthcare settings. These potential findings would be useful to support the visibility of the evidence of the effects of mindfulness-based interventions on psychological disturbances in AD caregivers and NPSs associated with AD, which could contribute to the engagement of stakeholders in healthcare.

Nevertheless, this study presents several limitations and strengths that should be acknowledged. One important limitation is that caregivers randomly allocated to the intervention group will receive an informative talk about mindfulness, particularly about the MBHC program, before starting the study. After this talk, caregivers will decide whether they want to continue in the study. Although this may imply a potential selection bias by compromising the randomization process of the study, we hope to minimize this shortcoming by adequately informing potential participants about the project through the informed consent form and the information sheet during the recruitment period. Regarding the control group, it is probable that they perform social, leisure, and/or recreational activities, including, yoga, relaxation techniques, etc., which means intervention biases can easily occur. However, we will collect information about any activities practiced regularly during leisure time to control for the likely influence of these variables. Concerning the instrument for measuring executive functions, the FAB, we used the Spanish version validated in people affected by Parkinson’s disease. It is known that the FAB has been validated in other populations with neurodegenerative diseases, including AD, and therefore, we assume that the version used in this study is appropriate for the assessment of our AD participants. Similarly, although pending the Spanish normative values, previous studies conducted in general population samples have found that the FAB is a suitable test for measuring executive performance in healthy individuals. Another limitation is that the main outcome variables will not be assessed using diagnostic tools but rather screening measures to identify the presence of symptoms or fulfilment of diagnostic criteria. The HAD scale and the NIP-Q are widely and easily used tools to detect anxiety and depression, respectively, in both medical and community populations [55], including caregivers [56], and NPSs that occur in AD [57]. Finally, although a likely dropout rate was calculated when designing this clinical trial, we are aware that dropouts may occur during the study, compromising the validity of the results. Another strategy for reducing the dropout rate that can be adopted is that participants who do not attend the post-intervention assessment at 8–10 weeks (T1) will be re-called at the follow-up assessment at 20–22 weeks (T2) to avoid missing data.

This study also presents several strengths. The design and tools we propose for this study have been based on previous similar randomized controlled clinical trials. The procedures will be carefully performed by a multidisciplinary professional group highly qualified in mindfulness-based interventions and care of neurological patients, including people suffering from AD. Thus, we believe that this study will yield accurate and useful information, thereby providing more convincing evidence about mindfulness-based interventions. Moreover, compared to earlier similar studies, our study adopts new approaches to caregiving problems in AD. Firstly, the originality of our study lies in the fact that it is a randomized clinical trial that seeks to assess in parallel the effects of a mindfulness-based intervention in the short- and medium-term over around 6 months (i.e., 20–22 weeks) on caregivers’ anxiety and depression and on the presence of NPSs in people diagnosed with AD. Although the intervention is specifically aimed at caregivers, this study will allow us to determine the relationship between NPSs in AD and caregiver outcomes. Secondly, this study intends to approach caregiving problems in AD from a different perspective focused on occupational performance and satisfaction, with the intention of improving meaningful participation in the daily living of both people affected by AD and their caregivers. In terms of feasibility, it should be noted that this study offers several advantages. For example, the intervention proposed will be carried out in groups of around 15 people who can be attended simultaneously, thereby being a low-cost community care program for caregivers. Working in groups will improve adherence to the intervention, favor social interaction between participants, and help them to restore their confidence and self-esteem. In addition, the exercises and techniques of the program are easy to understand, manage, and use for daily living, and more specifically, for activities related to the care of people with AD. Accordingly, the MBHC program could be a suitable community care program aimed at improving mental health issues that commonly affect the caregivers of people with AD.

## 4. Conclusions

This randomized controlled trial may represent a step forward in the treatment and prevention of mental health disturbances in caregivers of people suffering from AD. To our knowledge, this will be the first study to examine a mindfulness-based intervention’s effect on health outcomes in caregivers in parallel with NPSs of AD, a main determinant of caregivers’ burden. Importantly, this dual approach will bring benefits to both caregivers and people affected by AD. Moreover, the MBHC program is specially designed for personal use during daily living to help caregivers improve their occupational performance and participation. In light of the available evidence on mindfulness, for this study, we propose an objective and reliable methodology using statistical and research procedures to address the limitations of the study design and validate previous findings. As such, this research proposal attempts to methodologically reinforce research on mindfulness-based interventions. Thus, we hope that our findings can constitute a suitable rationale for replication in further randomized controlled longitudinal studies with large sample sizes. Taking into account the potentiality of mindfulness for the treatment of mental health issues by cultivating a healthy mind and improving wellbeing [58], the MBHC program offers a new and excellent opportunity to provide an innovative technique for reducing anxiety and depression in AD caregivers. Of note, the findings of this study, if confirmed, should have implications for future healthcare strategies to promote MBHC as a community care program to improve the quality of life and wellbeing of caregivers.

## Figures and Tables

**Table 1:** Structure of sessions in the Mindfulness-Based Health Care program.

Session	Main Topic	Content	Home Practice Exercises
1	Cultivating presence	Breathing exercise and conscious posture. Visualization meditation. Written exercise: perception of current personal situation and expectations of the course. Introductions and orientation of the course. Mindful eating exercise: eating a piece of fruit. ○ Sharing experiences with the group. Short practice exercise: “The pause”. Meditation: “Total relaxation” (body scan). ○ Sharing experiences with the group. Instructions on how to do home practice exercises.	Meditation: “Total relaxation” (body scan), once a day. Short practice exercise: “The Pause”, 3 times a day. Mindfulness in an activity of daily life, once a day
2	Awakening joy	Mindful movements. Meditation: “The Inner Smile”. ○ Sharing experiences (including both session and home practice exercises) with the group. Short practice exercise: “10 breaths for happiness”. Calendar of pleasant events. Meditation: “Total Relaxation” (body scan). ○ Sharing experiences with the group. Instructions on how to do home practice exercises.	Meditation: “The inner smile” and “Total relaxation” (body scanner), practiced alternately for 6 days. Short practice exercise: “10 Breaths for Happiness”, once a day. Mindfulness in one activity of daily life, once a day. Filling in a calendar of pleasant events.
3	Turning off the indoor radio	Mindful movements. Walking meditation. Meditation on thoughts. ○ Sharing experiences (including both session and home practice exercises) with the group. Short practice exercise: “The standing mountain”. Sitting meditation: “The inner island”. ○ Sharing experiences with the group. Instructions on how to do home practice exercises.	Sitting meditation, once a day. Filling in calendar of pleasant events. Short practice exercise: “The standing mountain”.
4	Cultivating happiness	Mindful movements. Meditation: “Happiness”. ○ Sharing experiences (including both session and home practice exercises) with the group. Short practice exercise: “The standing mountain”. Silent meditation. ○ Sharing experiences with the group. Instructions on how to do home practice exercises.	Meditation: “Vital Air” and “Happiness”, practiced alternately for 6 days. Exercise: “Smile when you wake up”, daily. Written exercise: “Moments of happiness”, once a day.
5	Awakening compassion	Mindful movements. Meditation: “Unconditional love for oneself”. ○ Sharing experiences (including both session and home practice exercises) with the group. Self-love practice: writing a love letter to oneself. Silent meditation. ○ Sharing experiences with the group. Instructions on how to do home practice exercises.	Meditation: “Unconditional love for oneself” and “Silent meditation”, practiced alternately for 6 days. Performing one self-care activity, daily.
6	Taking care of my thoughts and emotions	Meditation: “Vital Air”. ○ Sharing experiences (including both session and home practice exercises) with the group. Meditation: “Your loving kindness”. Written exercise: what qualities have you found in yourself? ○ Sharing experiences with the group. Silent meditation. ○ Sharing experiences with the group. Instructions on how to do home practice exercises.	Meditation: “Your loving kindness”, once a day. Exercise: “Love and compassion” for the acceptance of thoughts and feelings, once a day.
7	Cultivating my well-being	Mindful movements. Meditation: “Pebbles”. ○ Sharing experiences (including both session and home practice exercises) with the group. Walking meditation: “Treasure hunt”. Written exercise: “Wish list”. ○ Sharing experiences with the group. Instructions on how to do home practice exercises.	Designing a weekly meditation plan using any of the meditations practiced in the previous weeks. Performing the meditation once a day. Selecting one of the wishes from the list developed in class and creating an action plan to fulfil it, once a day.
8	Circle of care	Mindful movements. Meditation: “Jackal-Giraffe”. ○ Sharing experiences (including both session and home practice exercises) with the group. Written exercise: end-of-program letter ○ Sharing experiences with the group. Meditation: “Unconditional love”. Farewell. ○ Sharing experiences with the group. Instructions on how to do home practice exercises.	

**Table 2:** Summary of the data collection for the ATENEA study.

Outcome Measures	Participants		Measurement Method
	Caregiver	Person with AD	
Main outcomes			
Anxiety	x		HAD scale [36]
Depression	x		HAD scale [36]
Neuropsychiatric symptoms		x	NPI-Q [37]
Health			
Severity of cognitive impairment		x	GDS [38]
Cognitive function		x	MMS [39]
Executive functions	x	x	FAB-E [40,41]
Functional capacity		x	DAD-E [42,43]
Burden	x		ZBI [44,45]
Quality of life	x	x	SF-36 questionnaire [46]; QoL-AD [47]
Occupational balance	x		OBQ-E [48]
Self-compassion	x		SCS [49]
Psychological well-being	x		PWBS [50]
Caregiving			
Kinship	x		ad hoc questionnaire
Cohabitation	x		ad hoc questionnaire
Time as caregiver (mo.)	x		ad hoc questionnaire
Caregiving time (h/d)	x		ad hoc questionnaire
Domestic help	x		ad hoc questionnaire
Help for caregiving	x		ad hoc questionnaire
Quality of personal relationship before the diagnosis of AD	x		ad hoc questionnaire
Reduction in workdays	x		ad hoc questionnaire
Sociodemographics			
Age (in years)	x	x	ad hoc questionnaire
Sex	x	x	ad hoc questionnaire
Education level	x	x	ad hoc questionnaire
Marital status	x	x	ad hoc questionnaire
Occupation	x	x	ad hoc questionnaire
Work status	x	x	ad hoc questionnaire
Number of people cohabiting	x	x	ad hoc questionnaire

Abbreviations: ATENEA, ATENción plena en Enfermedad de Alzheimer; AD, Alzheimer’s disease; HAD, Hospital Anxiety and Depression; NPI-Q, Neuropsychiatric Inventory Questionnaire; GDS, Global Deterioration Scale; MMS, Mini Mental State; FAB-E, Frontal Assessment Battery (Spanish version); DAD-E, Disability Assessment for Dementia (Spanish version); ZBI, Zarit Burden Interview; QoL-AD, Quality of Life-Alzheimer’s Disease; OBQ-E, Occupational Balance Questionnaire (Spanish version); SCS, Self-Compassion Scale; PWBS, Psychological Well-Being Scale; mo., months, h/d, hours per day.

## Data Availability

Study data accessibility will be restricted, although data will be available on request. All requests will be reviewed by the principal investigator (A.S.-P.) and the research team and will require a data transfer agreement.

## Supplementary Materials

The following supporting information can be downloaded at: https://www.mdpi.com/article/10.3390/healthcare10030542/s1. File S1: Encuesta de opinión a cuidadores de personas con enfermedad de Alzheimer sobre la docencia en el curso.
