# Supplementary material for: The ATENción Plena en Enfermedad de Alzheimer (ATENEA—Mindfulness in Alzheimer’s Disease) Program for Caregivers: Study Protocol for a Randomized Controlled Trial
Source: Healthcare (Basel). 2022 Mar 15;10(3):542. doi: 10.3390/healthcare10030542 (PMC8955639; doi:10.3390/healthcare10030542)
Supplement: Supplementary file 1 [file healthcare-10-00542-s001.zip › healthcare-1573035-supplementary.pdf]

ENCUESTA DE OPINIÓN A CUIDADORES DE PERSONAS CON ENFERMDAD DE ALZHEIMER SOBRE LA DOCENCIA EN EL CURSO:
PROGRAMA DE CUIDADO DE LA SALUD BASADO EN MINDFULNESS

Indique su grado de acuerdo con las siguientes afirmaciones:

A: Totalmente en desacuerdo
B: En desacuerdo
C: Ni de acuerdo ni en desacuerdo
D: De acuerdo
E: Totalmente de acuerdo
F: No contesta

	A	B	C	D	E	F
El profesor proporciona información clara sobre los contenidos del curso: objetivos, programa, etc…						
El profesor explica de forma clara y organizada						
La forma de impartir la clase el profesor consigue motivarme y despertar el interés por el curso						
Las prácticas realizadas durante el curso y en casa ayudan a comprender mejor los contenidos teóricos						
El profesor ha resuelto adecuadamente las dudas que se han planteado en clase						

Indique su satisfacción:

0: Totalmente insatisfecho
10: Totalmente satisfecho

	0	1	2	3	4	5	6	7	8	9	10
Mi nivel de satisfacción con la labor del profesor											
Mi nivel de satisfacción con el curso											

Por favor, escriba aquí las sugerencias o comentarios que desee realizar:

____

____

____

____

____

____

____

____
